# Imaging Findings of a Typical Hemorrhagic Hepatic Cyst

**DOI:** 10.5334/jbr-btr.1200

**Published:** 2016-10-26

**Authors:** Guillaume Verlynde, Patrick Mailleux

**Affiliations:** 1 Department of Radiology, Clinique St-Luc, Bouge (Namur), Belgium

**Keywords:** Hemorrhagic hepatic cyst, Cystadenoma, Ultrasound, CT, MRI

A 72-year-old woman was referred to our hospital for a persistent right upper abdominal pain after a fall three weeks prior. An ultrasound was performed. This exam demonstrated a heterogeneous cystic mass in the right hepatic lobe, just under the right hemidiaphragm, measuring 11 cm in diameter, with multiple mobile septa (Figure 1) and a hyperechogenic inferior compartment. There was no calcification and the Color Doppler US showed no Doppler signal.

**Figure 1:**
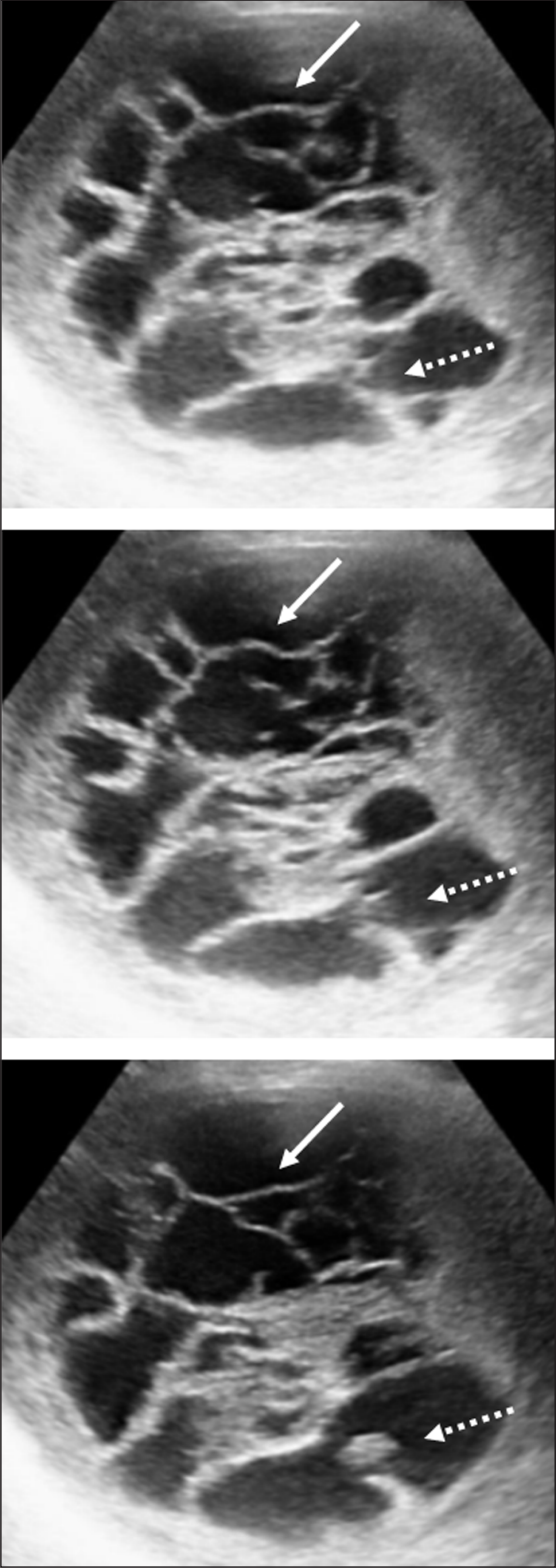


A CT scan without and after contrast enhancement showed a voluminous cystic lesion of 13 × 11 × 11 cm, with regular contours occupying the right hepatic dome. However, the multiple hyperechogenic mobile septa detected on ultrasonography (Figure 2a) were not visualized, even with enhanced CT (Figure 2b). As in the ultrasound examination, there was a non-enhancing gravity-dependent hyperdensity (33 Hounsfield unit), corresponding to a blood clot (Figure 2b). These findings are typical of a hemorrhagic hepatic cyst (HHC).

**Figure 2:**
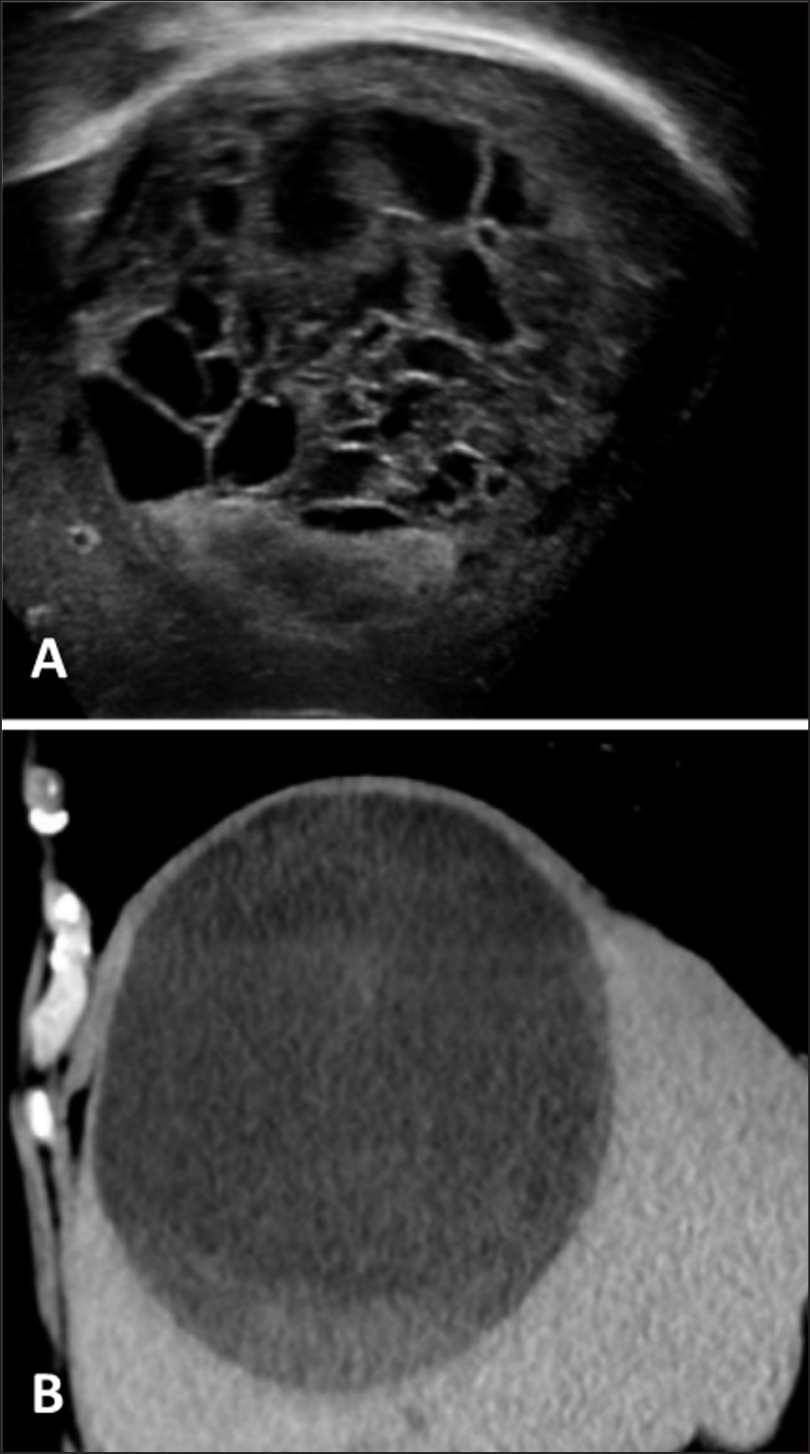


An MRI performed two months after initial assessment demonstrated a significant volume decrease of this hemorrhagic cyst. Changes of the cyst content signal confirmed its hemorrhagic nature: hyperintense on T1-weighted sequences without and with fat saturation, with a fluid-fluid level (Figure 3a). On T2-weighted images, the major portion in the superior part showed an intermediate signal, whereas the lower part is hypointense, suggesting a blood clot. The cyst is surrounded by a rim of hypointensity on T2- and T2*-weighted sequences corresponding to haemosiderin (Figure 3b). After the administration of Gadolinium, there is no enhancement.

**Figure 3:**
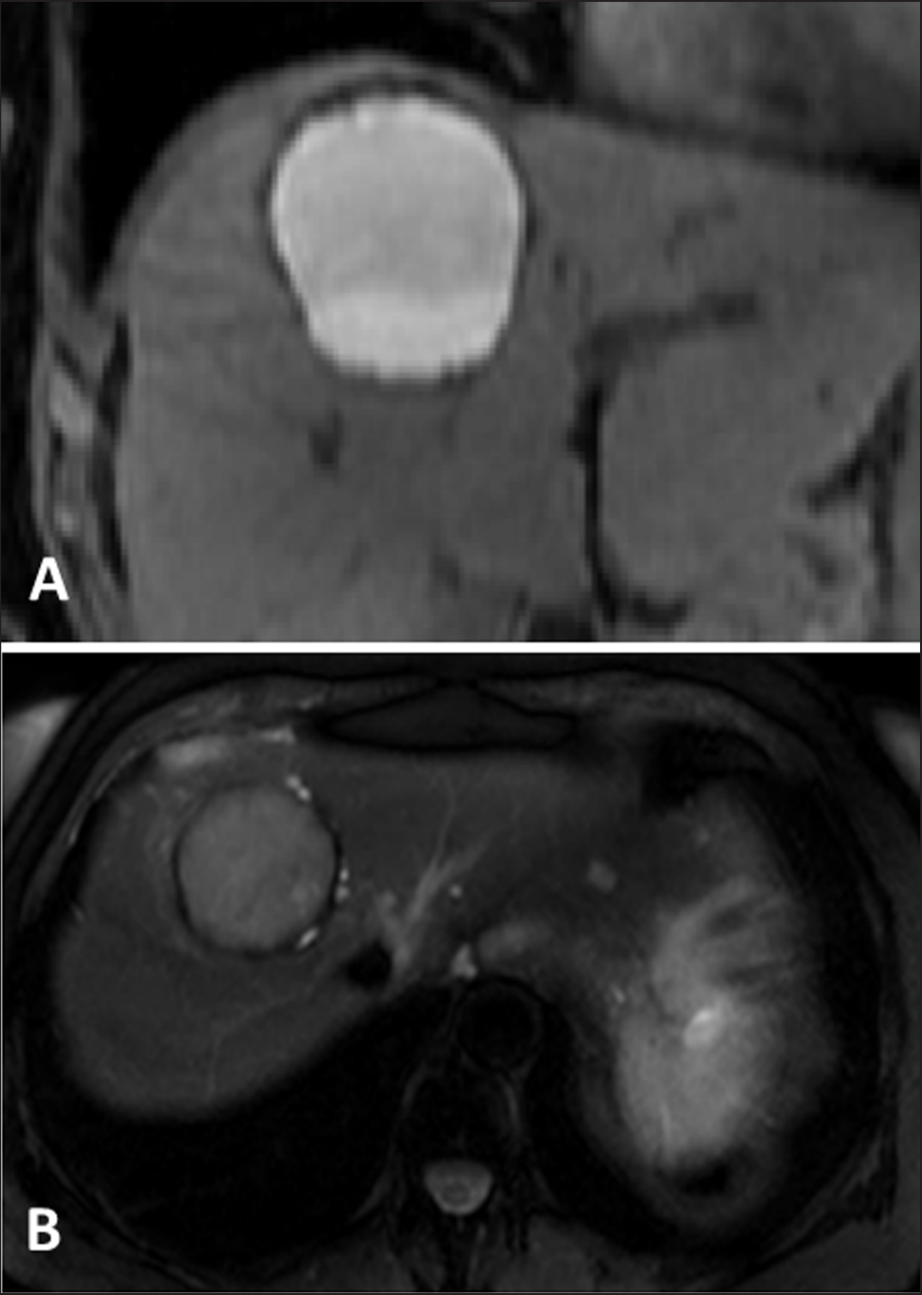


## Comment

Hemorrhage into a simple hepatic cyst results in the development of a complex cystic lesion, which may be indistinguishable from a cystic tumor and, as a consequence, may result in a broad differential diagnosis, e.g. simple cysts, infectious or inflammatory conditions (hepatic abscess, amoebic hepatic abscess, hepatic hydatid cyst), neoplastic or tumorous (biliary cystadenoma or cystadenocarcinoma, cystic hepatic metastases, cystic cavernous haemangioma), and others (liquified hepatic haematoma, infected cyst, biloma).

During physiological clot lysis the presence of multiple septa and vegetations, mural nodules, and thickened cyst wall may be observed [1]. A liquid-liquid level is possible. The principal challenge is to differentiate simple hepatic cyst complicated by intracystic hemorrhage from cystic neoplasms. US imaging plays an important role in achieving a correct diagnosis of a hemorrhagic cyst when it demonstrates the mobility of the septa. These irregular mobile septa visualized on US imaging are not found on CT. While cystadenocarcinoma is characterized by thick septa visible both on US and on CT. This discordance between US and CT findings is an important element for differential diagnosis between HHC versus cystadenocarcinoma. However, a cystic tumorous lesion may show similar findings when it is mainly composed of a cyst with prominent intracystic hemorrhage.

Contrast-enhanced US improve the sensibility showing microbubbles oozing from the cyst wall into the cyst cavity when there is intracystic bleeding and revealing no enhancement of the intracystic structures, then suggesting intracystic clot. Signal intensities and the MRI characteristic on follow-up examinations may be also helpful to demonstrate the hemorrhagic nature of a cystic lesion and to differentiate it from other mucinous cyst on MRI.

Imaging may fail to differentiate hepatic simple cysts from malignant or premalignant mucinous cystic lesions such as biliary cystadenomas. Hepatic simple cysts can be treated conservatively, whereas malignant or premalignant cysts require complete resection. The concentration of TAG-72 in cyst fluid accurately differentiate simple cyst from mucinous cysts requiring complete resection [2].

The therapeutic approach for HHC has varied from simple observation to more invasive approaches. HHC that are mildly symptomatic or asymptomatic can be initially treated by conservative approach with a series of imaging control to monitor the evolution. If there is a deterioration or persistence of symptoms, a laparoscopic or open de-roofing with cyst wall biopsy or enuclation is indicated. When there is spontaneous resolution, a follow-up by US is sufficient.

In conclusion, hemorrhagic hepatic simple cyst represent a difficult diagnosis because of its imaging similarities with other cystic or mucinous hepatic lesions. The mobility of septa on US imaging, when not found on CT images, is suggestive of an hemorrhagic content. Enhancement of thick septa, mural nodules or thickened wall are typically found in malignant lesions. MRI signal intensities help to differentiate hemorrhagic content of a cyst from other mucinous cyst. All these elements may lead to the accurate diagnosis and guide appropriate therapeutic decisions.
